# Identification and Characterization of Holin-like Protein ORF70 from Cyanophage MaMV-DC

**DOI:** 10.3390/md24010014

**Published:** 2025-12-26

**Authors:** Lihui Meng, Yi Wu, Jiahao Xu, Jiarui Zhang, Zhiyong Zhang, Chen Wang

**Affiliations:** Laboratory of Aquatic Parasitology and Microbial Bioresources, School of Marine Science and Engineering, Qingdao Agricultural University, Qingdao 266237, China; who79063@163.com (Y.W.); xjh200682@126.com (J.X.); 19859516223@163.com (J.Z.); 15923499167@163.com (Z.Z.);

**Keywords:** holin-like protein, cyanophage MaMV-DC, ORF70, bacteriolytic activity

## Abstract

In this study, we characterized the holin-like protein ORF70 from the cyanophage MaMV-DC, offering valuable insights into its role in phage-mediated host cell lysis. ORF70 shares key features with class III holins, such as a hydrophobic transmembrane domain and membrane-associated localization, which are crucial for its bacteriolytic activity. Subcellular localization studies suggested its association with the membrane, supporting its classification as a holin-like protein. Overexpression of ORF70 in *E. coli* resulted in significant growth inhibition, increased β-galactosidase leakage, and visual confirmation of cell death through live/dead staining. Additionally, ORF70’s sensitivity to the energy toxin 2,4-dinitrophenol (DNP) further indicated its holin-like activity by promoting membrane depolarization. Transmission electron microscopy and Gram staining revealed characteristic morphological changes in *E. coli* cells, including membrane disruption, consistent with damage caused by holins. These results suggest that ORF70 acts as a holin-like protein that disrupts the host membrane, leading to bacterial cell death. Our study provides evidence supporting the holin-like activity of ORF70 from cyanophage MaMV-DC. This research significantly enhances our understanding of phage-host interactions and opens new avenues for developing phage-based therapies, offering promising alternatives to traditional antibiotics amidst the growing challenge of antibiotic resistance.

## 1. Introduction

Cyanobacteria, present on Earth for more than 3.5 billion years, are found extensively in aquatic ecosystems. As a key group of photosynthetic bacteria, they play a vital role in oxygen production, nitrogen and carbon dioxide fixation, making them an ideal model for exploring the integration of carbon and nitrogen metabolic processes [1]. Cyanophages, viruses that target cyanobacteria, play a crucial role in shaping host communities, food webs, and nutrient cycles [2]. They influence the regulation of cyanobacterial blooms by inducing cell lysis and altering community composition [3,4]. These viruses typically employ a holin-endolysin system, comprising two adjacent genes encoding holin and endolysin proteins, to lyse their host cells [5]. Holin, a small hydrophobic membrane protein, enables the transport of endolysin into the periplasm, where it breaks down the peptidoglycan layer, causing the host cell to lyse in the later stages of infection [6,7,8,9,10,11]. The presence of this system has been identified in various cyanophages, including the tailless PaV-LD and long-tailed phages such as Mwe-Yong 1112-1 and VB_MelS-Me-ZS1. Notably, in PaV-LD, the holin and endolysin proteins work synergistically to exhibit robust bacteriolytic activity [12,13]. Another strategy of bacteriophage-mediated bacterial lysis relies on lyases to inhibit the synthesis of peptidoglycan or hydrolyze the host cell wall. Interestingly, the *Myoviridae* cyanophage lacks the conventional holin-endolysin lysis system and instead possesses one or two lyases [14]. Studies have shown that the two independent lysis-related genes of cyanophage MaMV-DH01 also exhibit strong lytic activity [15]. Therefore, understanding the diverse lysis mechanisms of cyanophages is important for advancing our understanding of cyanophage–host interactions and may provide a theoretical basis for exploring their potential roles in cyanobacterial bloom mitigation.

Holins are crucial proteins for host cell lysis, typically encoded by double-stranded DNA phages [16,17]. During the late stages of infection, holins create non-specific pores or lesions in the host’s cytoplasmic membrane, allowing endolysins to access the cell wall and induce lysis [18]. While holins range in size from 49 to 210 amino acids, they share several key features [16]: (1) their genes are usually located near endolysin genes, (2) they contain at least one transmembrane α-helical sequence [17]; and (3) they have a charged, hydrophilic C-terminal domain. Holins can be classified into three types based on their protein structure and the number of transmembrane domains (TMDs): Class I (longer than 95 aa, 3 TMDs), Class II (65–95 aa, 2 TMDs), and Class III (1 TMD) [16,17].

Numerous endolysins and holins from phages have been explored as potential antibacterial agents, including those derived from Staphylococcus aureus phage GH15, Salmonella phage P22, and Streptomyces avermitilis phage phiSASD1 [19,20,21]. However, research on homologous proteins from cyanophages remains limited. In this study, we characterized the gene (ORF70) encoding a holin-like protein from MaMV-DC using bioinformatics tools. Molecular experiments suggest the bacteriolytic activity of ORF70. Based on these results, we propose that ORF70 may contribute to the development of novel antibacterial therapies.

## 2. Results

### 2.1. Identification and Sequence Analysis of ORF70

**Figure 1:**
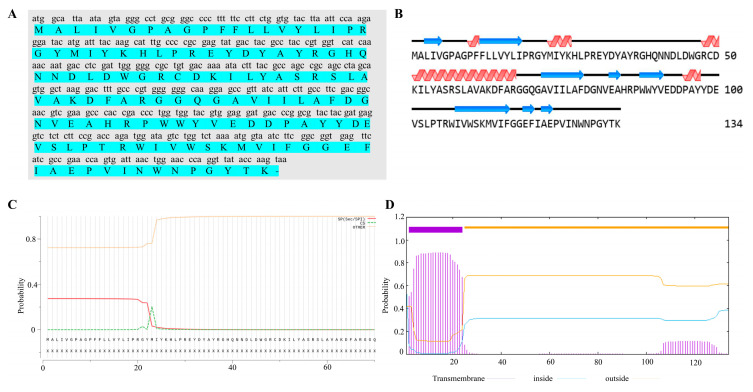
Structural characteristics and evolutionary analysis of ORF70 protein sequence. (**A**) The nucleotide sequence of ORF70 and its corresponding amino acid sequence are shown, with the amino acid sequence highlighted in green. (**B**) Secondary structure prediction analysis of ORF70. The α-helices are represented by red helical shapes, while the β-sheets are depicted as blue arrows. (**C**) Signal peptide prediction for ORF70. The x-axis represents the amino acid sequence of ORF70, while the y-axis indicates the probability of signal peptide presence, ranging from 0 to 1. The red line represents the probability of the Sec/SPI pathway (SP), indicating the likelihood of the presence of a signal peptide. The horizontal thresholds indicate the cutoff values for signal peptide prediction, with values above 0.5 suggesting a signal peptide. (**D**) Prediction and analysis of transmembrane structures. Transmembrane helix prediction (TMHMM) showing the predicted transmembrane domain spanning residues ~2–24, with inside/outside probabilities plotted along the sequence.

ORF70 of MaMV-DC has been identified as a putative holin-like protein. The ORF70 gene was 405 bp in length and encodes a protein of 134 amino acids (aa), with a calculated molecular mass of 15.4 kDa and a theoretical isoelectric point (pI) of 5.86. Secondary structure analysis of the ORF70 protein reveals the presence of four α-helices and eight β-strands (Figure 1B). According to SignalP 4.0 software, ORF70 lacks a signal peptide (Figure 1C). Structural predictions further suggest that ORF70 contains a single hydrophobic transmembrane domain (TMD) and an extracellular C-terminal region, with the transmembrane helix spanning residues 2–24 (Figure 1D). Based on its predicted structural characteristics, ORF70 was considered a holin-like protein with features consistent with class III holins.

### 2.2. Subcellular Localization of ORF70

**Figure 2:**
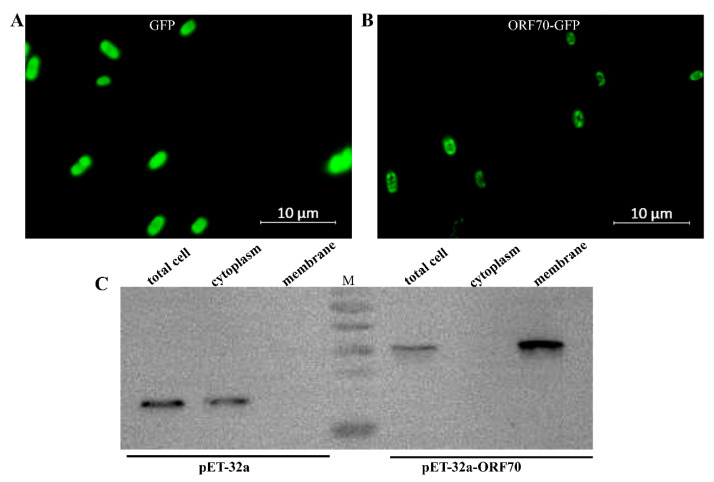
Localization of ORF70 protein in *Escherichia coli*. (**A**) Fluorescence image of *E. coli* (DE3: pET32a-GFP) expressing GFP. (**B**) Fluorescence image of *E. coli* (DE3: pET-32a-ORF70-GFP) expressing ORF70-GFP. (**C**) The subcellular localization of ORF70 was analyzed using Western blotting.

The ORF70 protein has been proposed as a membrane-associated holin-like protein, though this hypothesis has yet to be substantiated through biochemical analysis. To investigate its properties, recombinant ORF70, fused with C-terminal sGFP (designated ORF70-G), was expressed in *E. coli* using the IPTG-inducible plasmid pET-32a-ORF70-GFP (IPTG, isopropyl-β-D-thiogalactoside, which activates the lac operon, enabling controlled recombinant protein expression in our experiments). The results indicate that the fluorescence signal in the control strain expressing GFP is evenly distributed (Figure 2A), whereas the fluorescence signal in BL21 cells expressing the ORF70-GFP fusion protein is faintly localized along the cell periphery (Figure 2B). This localization pattern is consistent with the expected distribution of a membrane-associated protein, but further confirmation through membrane fractionation experiments is required to provide stronger evidence for its membrane localization. To further confirm the membrane localization of ORF70, the protein was expressed as a C-terminal His-tag fusion (ORF70-His), and cytoplasmic and membrane fractions were isolated for Western blot analysis. The results showed that ORF70-His was detected in the membrane fraction (41.2 kDa), while the control protein (32a-His) was predominantly located in the cytoplasm (33.8 kDa) (Figure 2C). These findings provide supporting evidence for the classification of ORF70 as a membrane-associated protein.

### 2.3. Analysis of Energy Toxin Test

**Figure 3:**
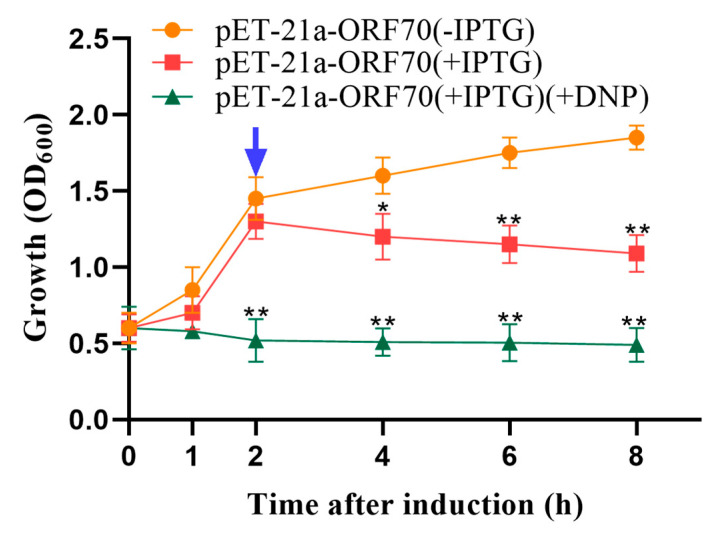
Effect of 2,4-dinitrophenol (DNP) on the growth of DE3: pET-21a-ORF70. After 25 min of IPTG induction, DNP was added to a final concentration of 10 mmol/L. The blue arrow indicates that DNP triggers ORF70 induced depolarization of the cell membrane 2 h in advance. The orange line represents the uninduced DE3: pET-21a-ORF70 (–IPTG), the red line represents the IPTG-induced DE3: pET-21a-ORF70 without DNP (+IPTG), and the green line represents the IPTG-induced DE3: pET-21a-ORF70 with DNP (+IPTG, +DNP). Expression of ORF70 results in growth inhibition of *E. coli*, which occurs earlier and is more pronounced in the presence of DNP. The “*” (*T*-test, *p* < 0. 05) and “**” indicates statistical significance (*T*-test, *p* < 0. 01).

Phage holins proteins induce bacterial lysis by depolarizing the cell membrane. Compounds such as 2,4-dinitrophenol (DNP) and other energy-toxic agents promote membrane depolarization, causing membrane damage to form in advance. Therefore, sensitivity to DNP serves as a key indicator of holin activity. Our results demonstrated that 10 mmol/L DNP induced premature membrane depolarization, enhancing the growth inhibition effect of ORF70 in *E. coli*. Specifically, the addition of DNP accelerated the reduction in cell density observed within one hour of IPTG induction in the DE3: pET-21a-ORF70 strain (*p* < 0.01; Figure 3). These findings confirm that ORF70 is sensitive to DNP and induces early membrane depolarization, supporting the classification of ORF70 as a holin-like protein.

### 2.4. Inhibitory Effect of ORF70 on E. coli Growth

**Figure 4:**
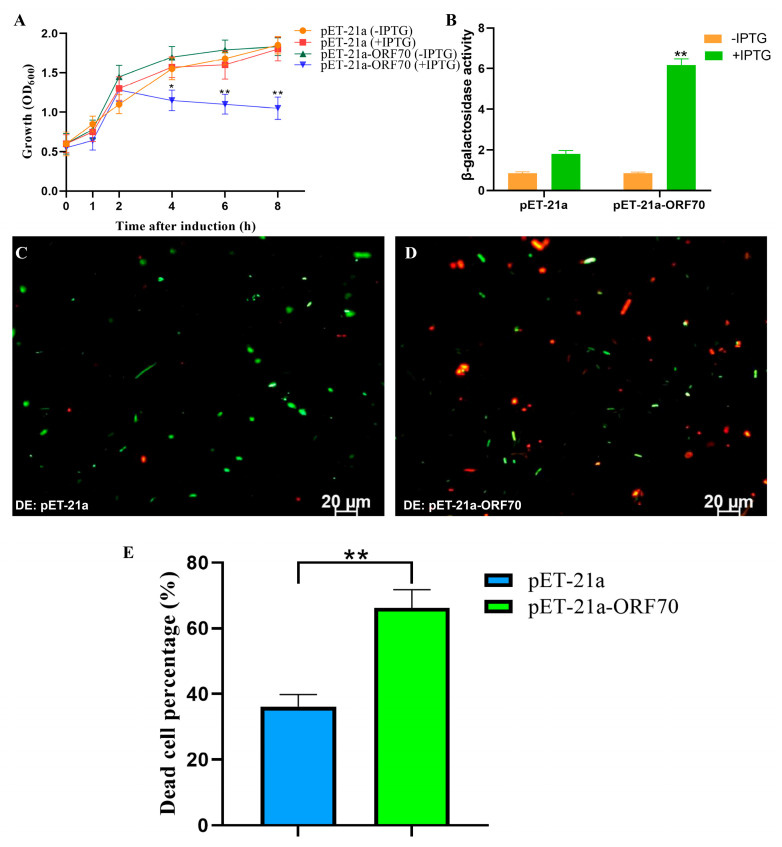
Effect of ORF70 on the growth activity of *E. coli*. (**A**) The growth curves of induced and uninduced strains (DE3: pET-21a and DE3: pET-21a-ORF70). (**B**) The β-galactosidase activity was determined for extracellular supernatant from induced strains. (**C**) Fluorescent staining of induced DE3: pET-21a. (**D**) Fluorescent staining of induced DE3: pET-21a-ORF70. The cells with green fluorescence represent living bacterial cells, and those with red fluorescence represent dead cells. (**E**) The percentage of dead cells of different *E. coli* strains. The “*” (*T*-test, *p* < 0. 05) and “**” indicates statistical significance (*T*-test, *p* < 0. 01).

To assess whether ORF70 exhibits the expected lytic activity of a Holin, we evaluated its expression and impact on *E. coli* growth. As shown in Figure 4A, the OD600 of *E. coli* strain DE3: pET21a-ORF70 decreased significantly after 2 h of IPTG induction, indicating a bactericidal effect of ORF70. Additionally, the β-galactosidase assay revealed elevated extracellular β-galactosidase levels upon ORF70 expression, suggesting that ORF70 induces cell membrane damage, leading to bacterial growth inhibition (*T*-test, *p* < 0.01, Figure 4B). Furthermore, live-dead staining performed after 2 h of IPTG induction showed red fluorescence in *E. coli* cells expressing ORF70, indicating cell death, in contrast to the control strain (Figure 4C,D and Figure S1). Quantitative analysis of live/dead staining results showed that, compared with the DE3: pET-21a control group, the DE3: pET-21a-ORF70 strain exhibited a significantly higher proportion of dead cells (*p* < 0.01; Figure 4E). Collectively, these results demonstrate that ORF70 not only inhibits *E. coli* growth but also causes membrane damage, leading to bacterial cell death.

### 2.5. ORF70 Exerted Bacteriolytic Activity by Causing Membrane Damage

**Figure 5:**
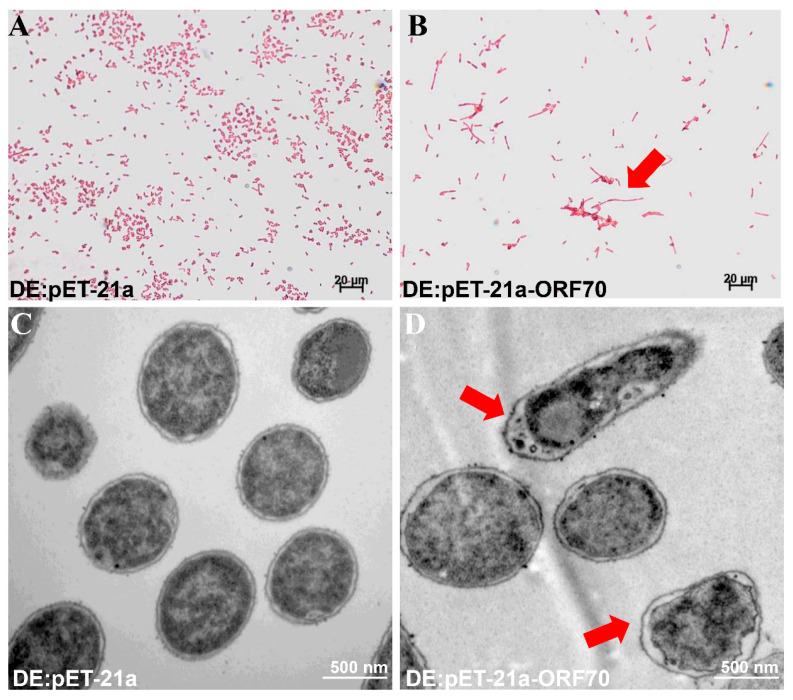
Effect of ORF70 on *E. coli* cell morphology. (**A**) Gram staining of induced DE3: pET-21a. (**B**) Gram staining of induced DE3: pET-21a-ORF70. (**C**) Induced DE3: pET-21a under TEM. (**D**) Induced DE3: pET-21a-ORF70 under TEM. Red arrows in panels (**B**,**D**) indicate morphological changes.

To investigate the mechanism behind the growth inhibition observed in *E. coli*, we examined the morphological changes induced by ORF70 overexpression using Gram staining and transmission electron microscopy (TEM). *E. coli* cells expressing ORF70 showed blurred cell boundaries, slight shrinkage between the cell wall and membrane, and a lighter cellular appearance (Figure 5A,C). In contrast, control cells (DE3: pET-21a) maintained normal morphology, with intact membranes, clear cell walls, and dense cellular contents (Figure 5B,D). Taken together, the data suggest that ORF70 from MaMV-DC may be involved in membrane perturbation, supporting a potential holin-like function.

## 3. Discussion

In this study, we characterized the holin-like protein ORF70 from the cyanophage MaMV-DC, shedding light on its role in phage-mediated host cell lysis. Our findings contribute to a deeper understanding of the mechanisms by which holin proteins function in cyanophage biology, particularly in disrupting host cell membranes. This research provides crucial insights into the molecular processes underlying phage-induced lysis and may inform future efforts toward exploring biotechnological applications, such as the development of novel antibacterial agents based on holin proteins.

The protein encoded by ORF70 exhibits several key characteristics that are consistent with class III holins. Specifically, it contains a hydrophobic transmembrane domain, which is a defining feature of holins. The results from sequence analysis and structural prediction align with previous studies on holins, which typically possess a transmembrane α-helical sequence and a hydrophilic C-terminal domain [19,20]. Furthermore, the subcellular localization studies indicated that ORF70 is membrane-associated, supporting its classification as a holin-like protein. This membrane association is critical for its bacteriolytic activity, as holins function by disrupting the host cell membrane, creating lesions that facilitate the translocation of endolysins to the cell wall, leading to cell lysis [22,23,24].

The potential bacteriolytic activity of ORF70 was suggested by several assays, including growth curve measurements and β-galactosidase leakage assays. Overexpression of ORF70 in *E. coli* led to a significant reduction in bacterial growth, accompanied by increased β-galactosidase activity in the extracellular medium, a hallmark of membrane disruption. These findings are consistent with the expected mechanism of holins, where membrane damage leads to the leakage of intracellular contents [6,25]. Furthermore, the live/dead staining results provided visual confirmation of cell death in the bacterial cultures expressing ORF70, with a significant proportion of cells exhibiting red fluorescence, indicative of compromised membrane integrity [8,23,26].

The sensitivity of ORF70 to the energy toxin 2,4-dinitrophenol (DNP) further supports its holin-like activity. Holins are known to interact with energy toxins to promote membrane depolarization and facilitate their bacteriolytic function [9,27]. Our results demonstrated that the addition of DNP accelerated the growth inhibition in *E. coli*, suggesting that ORF70 induces membrane depolarization, a key step in the lytic process. This finding provides additional evidence for the function of ORF70 as a holin-like protein that contributes to bacterial cell death through membrane disruption.

Transmission electron microscopy (TEM) and Gram staining further revealed the morphological changes induced by ORF70. The *E. coli* cells expressing ORF70 exhibited significant alterations in cell morphology, including blurred cellular boundaries and membrane shrinkage. These changes are characteristic of membrane damage caused by holins [13]. The observed morphological defects are consistent with the known bacteriolytic activity of holins, which form pores or lesions in the host membrane, leading to cell death and the release of intracellular contents [13,26].

This study contributes to the expanding knowledge of cyanophage biology and the role of holins in phage-host interactions. While much of the existing research has focused on the holin-endolysin systems of bacteriophages infecting Gram-positive bacteria, fewer studies have explored the holins of cyanophages. Our findings provide functional characterization of ORF70 as a holin-like protein and suggest its potential role in phage-mediated host cell lysis. However, it should be noted that *Microcystis aeruginosa* FACHB-524 lacks an available genetic manipulation system, which currently prevents heterologous expression of ORF70 and further lysis assays in this cyanobacterial host. Consequently, its potential role in bloom mitigation or antimicrobial applications remains to be evaluated in future studies. Instead, the findings highlight the functional characteristics of ORF70 as a holin-like protein and its potential contribution to phage-mediated lysis. The identification of a holin-like protein in a cyanophage provides valuable insights that may inform future exploration of phage-based therapies, offering a potential alternative to traditional antibiotics, particularly in light of the growing issue of antibiotic resistance [12,28,29]. Future research should focus on the expression and functional analysis of ORF70 in cyanobacterial hosts, as well as the structural dynamics of ORF70 within the membrane and its interactions with other phage proteins, such as endolysins. Additionally, the ecological implications of holin activity in cyanophages, particularly their potential role in regulating cyanobacterial populations in natural environments, require further investigation.

## 4. Materials and Methods

### 4.1. Sequence Analysis

The protein sequence of ORF70 was predicted using the ExPASy Translate Tool (https://web.expasy.org/translate/, accessed on 20 October 2025). Protein similarity was assessed via BLASTP (v2.8) against the NCBI nr-database. Protein characteristics such as molecular weight, amino acid composition, and theoretical isoelectric point (pI) were determined using the ProtParam tool (https://web.expasy.org/protparam/, accessed on 20 October 2025). Signal peptides were identified using SignalP 5.0 (http://www.cbs.dtu.dk/services/SignalP/, accessed on 20 October 2025). The secondary structure was predicted using NOVOPRO (https://novopro.cn/tools/secondary-structure-prediction.html, accessed on 20 October 2025). The GenBank accession number of ORF70 from MaMV-DC was AGR48635.1.

### 4.2. Bacteria, Cyanobacteria, and Cyanophage Culture

The bacterial strains and plasmids used in this study are listed in Table 1. The ORF70 gene was expressed in *E. coli* BL21 (DE3) (Vazyme, Nanjing, China), which were grown in LB (Luria–Bertani) broth or agar at 37 °C. Ampicillin (100 μg/mL, Sangon Biotech, Shanghai, China) was added to the culture medium unless specified otherwise. *M. aeruginosa* FACHB-524 was cultured as previously described, and the cyanophage MaMV-DC was propagated using this strain as the host [15].

### 4.3. Plasmid Construction

Genomic DNA from cyanophage MaMV-DC was used as a template for PCR amplification with specific primers (Table 2) and high-fidelity DNA polymerase (Vazyme, Nanjing, China). The PCR products containing ORF70 were purified using a PCR Purification Kit (OMEGA, Norcross, GA, USA), and the target fragments were inserted into the expression vectors pET-21a (+) and pET-32a (+) using a One Step Cloning Kit (Vazyme, Nanjing, China). The resulting plasmids were introduced into competent *E. coli* BL21 (DE3) for expression. Additionally, we constructed a fusion plasmid, pET-32a-ORF70-GFP, to track the cellular localization of ORF70 in *E. coli*.

### 4.4. Localization of ORF70 in E. coli

The strains (DE3: pET32a-ORF70-GFP and DE3: pET32a-GFP) were cultured in LB broth with ampicillin. When the OD600 reached 0.6, IPTG was added to a final concentration of 0.1 mM, and the temperature was adjusted to 30 °C. After 4 h of induction, cells were harvested by centrifugation (10,000 rpm, 10 min), washed with PBS, and observed under a fluorescence microscope (ZEISS LSM900, Oberkochen, Germany). To further confirm ORF70 localization in *E. coli*, cell components were isolated for Western blotting. Membrane fractions were extracted using an *E. coli* membrane protein extraction kit (BestBio, Shanghai, China). The whole cell, cytoplasmic, and membrane fractions were analyzed by Western blotting. Proteins were transferred to a PVDF membrane (Millipore, Bedford, MA, USA) and detected with anti-His-tagged mAb (CST) and HRP-conjugated goat anti-mouse IgG (Beyotime, Shanghai, China). The bands were visualized using Pierce ECL Plus Western blotting Substrate (Thermo Fisher Scientific, Waltham, MA, USA). The empty pET32a plasmid served as the control.

### 4.5. Bacterial Growth Curve Assays

The optical density (OD600) of *E. coli* was measured to evaluate the effect of ORF70 from MaMV-DC, following the method described earlier [13]. Briefly, overnight cultures were diluted 1:100 into fresh medium and grown to an OD600 of 0.6. IPTG (1.0 mM) was then added, and OD600 readings were taken every 30 min. Strains without IPTG were used as negative controls, and the experiment was performed in triplicate with three replicates per treatment. Cell viability was determined using a live/dead fluorochrome dye kit (Mailian Biotechnology, Shanghai, China). Two hours after IPTG induction, the cells were stained following the manufacturer’s protocol. The microphotographs of each sample were taken under a fluorescence microscope (Leica DMB, Leica, Wiesbaden, Germany) with an N21 (BP 515-560) filter for green fluorescence and a CY5-T (BP 635/10) filter for red fluorescence. The number of dead bacteria was quantified from three independent experiments using ImageJ (2.x). The percentage of dead cells was calculated by comparing the number of dead fluorescence-positive cells to the total number of live and dead fluorescence-positive cells. Statistical analysis was performed using GraphPad Prism (version 8.0), and the results were plotted with appropriate error bars.

### 4.6. Toxin Assay for Energetic Toxins

Most holins are capable of depolarizing the cell membrane in the presence of certain energy toxins, such as 2,4-dinitrophenol (DNP) or potassium cyanide, leading to membrane damage and pore formation [30,31]. Therefore, sensitivity to DNP serves as a key indicator for holin activity. In this experiment, 2 mL of a 1 mol/L DNP stock solution was freshly prepared. BL21(DE3) cells transformed with the pET-21a-ORF70 plasmid (OD600 ~0.5) were induced with IPTG at a final concentration of 1 mmol/L, and the optical density (OD) was monitored. After 25 min of IPTG induction, DNP was added to a final concentration of 10 mmol/L (1:100 dilution), with a negative control group where DNP was omitted. The OD of the culture was continuously recorded for 120 min post-induction.

### 4.7. Measurement of β-Galactosidase Activity

To evaluate membrane integrity, 500 μL of the extracellular supernatant from the specified strains was mixed with 20 mM ONPG (O-nitrophenyl-β-D-galactopyranoside, TaKaRa, Dalian, China) to achieve a final volume of 100 μL. The mixture was incubated at 37 °C for 1 h, then 0.5 mol/L Na_2_CO_3_ was added to stop the reaction. The optical density (OD420) of the supernatant was measured to determine β-galactosidase activity. β-galactosidase assays were performed using three independent biological replicates, each measured in technical triplicate. The activity (U/mL) was calculated using the formula: (OD420·V)/(T·VS·0.0045), where V is the volume of the mixture (mL), T is the reaction time (minutes), vs. is the volume of the sample used to measure OD420 (mL), and 0.0045 is the extinction coefficient (mL/nmol) [13].

### 4.8. Morphological Observation of Bacteria

The bacteria were collected by centrifugation (5000× *g* for 5 min) and then washed three times with PBS (0.1 M). Gram staining was carried out using a Gram Stain Kit (Solarbio, Beijing, China) and examined under a light microscope (ZEISS LSM900, Germany). For transmission electron microscopy (TEM) analysis [32], the bacteria were fixed with 2.5% glutaraldehyde and then stained with 0.1% osmium tetroxide for 1 h. The bacteria were then dehydrated through a series of ethanol solutions (70%, 80%, 90%, 95%, and 100% *v*/*v*), with each step lasting 15 min at 25 °C. The samples were embedded in Epon 812, sectioned into ultrathin slices, and examined using a Hitachi H-7650 TEM to observe bacterial morphological changes. TEM observations were performed using samples from three independent biological replicates, and multiple fields of view were examined for each replicate.

### 4.9. Statistical Analysis

The *T*-test was performed using GraphPad Prism (version 8.0). Data are presented as mean ± standard deviation from at least three independent experiments, with statistical significance considered at a *p*-value of <0.05.

## 5. Conclusions

Our study provides evidence supporting the holin-like activity of ORF70 from cyanophage MaMV-DC. This research suggests potential avenues for the application of phage holins in antimicrobial therapy and offers insights into the molecular mechanisms underlying phage-induced cell lysis. Further investigations into the detailed mechanisms of holin function and the potential for phage-based antimicrobial agents may contribute to advancing this field.

## Figures and Tables

**Table 1:** Strains/cyanophage used in this study.

Strains/Cyanophages	Genotype or Relevant Properties	Source
*E. coli* DH5α	supE44, ΔlacU169 (φ80 lacZ ΔM15), hsdR17, recA, endA1, gyrA96, thi-1, relA1	Takara
*E. coli* BL21 (DE3)	*E. coli* B, F-, ompT, hsdSB, (rB-, mB-), dcm, gal, k(DE3) pRARE (CamR)	ZOMANBIO
DE3: pET-32a	Strains containing plasmid pET-32a	This lab
DE3: pET-32a-ORF70	Strains containing plasmid pET-32a-ORF70	This lab
DE3: pET-32a-GFP	Strains containing plasmid pET-32a-GFP	This lab
DE3: pET-32a-ORF70-GFP	Strains containing plasmid pET-32a-ORF70-GFP	This lab
DE3: pET-21a	Strains containing plasmid pET-21a	This lab
DE3: pET-21a-ORF70	Strains containing plasmid pET-21a-ORF70	This lab
cyanophage MaMV-DC	Wild-type, 169,223 bp dsDNA genome	This lab

**Table 2:** Primers used in this study.

Primer	Sequence (5′–3′)
pET-32a-ORF70-FW	gctgatatcggatccgaattcATGGCATTAATAGTAGGGCCTGC
pET-32a-ORF70-RV	ttgtcgacggagctcgaattcCTTGGTATAACCTGGGTTCCAGTTA
pET-21a-ORF70-FW	atgggtcgcggatccgaattcATGGCATTAATAGTAGGGCCTGC
pET-21a-ORF70-RV	ttgtcgacggagctcgaattcCTTGGTATAACCTGGGTTCCAGTTA
pET-32a-GFP-*NotI*-FW	tccgtcgacaagcttgcggccgcATGGTGAGCAAGGGCGAGGA
pET-32a-GFP-*NotI*-RV	tggtggtgctcgagtgcggccgcCTGTACAGCTCGTCCATGCCG

FW: sense primer; RV: antisense primer.

## Data Availability

The raw data supporting the conclusions of this article will be made available by the authors on request.

## Supplementary Materials

The following supporting information can be downloaded at: https://www.mdpi.com/article/10.3390/md24010014/s1, Figure S1: (A,B) Fluorescent staining of induced DE3: pET-21a. (C,D) Fluorescent staining of induced DE3: pET-21a-ORF70.
