# Supplementary material for: Identification and Characterization of Holin-like Protein ORF70 from Cyanophage MaMV-DC
Source: Mar Drugs. 2025 Dec 26;24(1):14. doi: 10.3390/md24010014 (PMC12843271; doi:10.3390/md24010014)
Supplement: Supplementary file 1 [file marinedrugs-24-00014-s001.zip › marinedrugs-4022851-supplementary.pdf]

marine drugs

Supplementary Material

Identification and characterization of holin-like protein ORF70 from cyanophage MaMV-DC

Lihui Meng [1,†,*], Yi Wu [1,†], Jiahao Xu [1], Jiarui Zhang [1], Zhiyong Zhang [1] and Chen Wang [1]

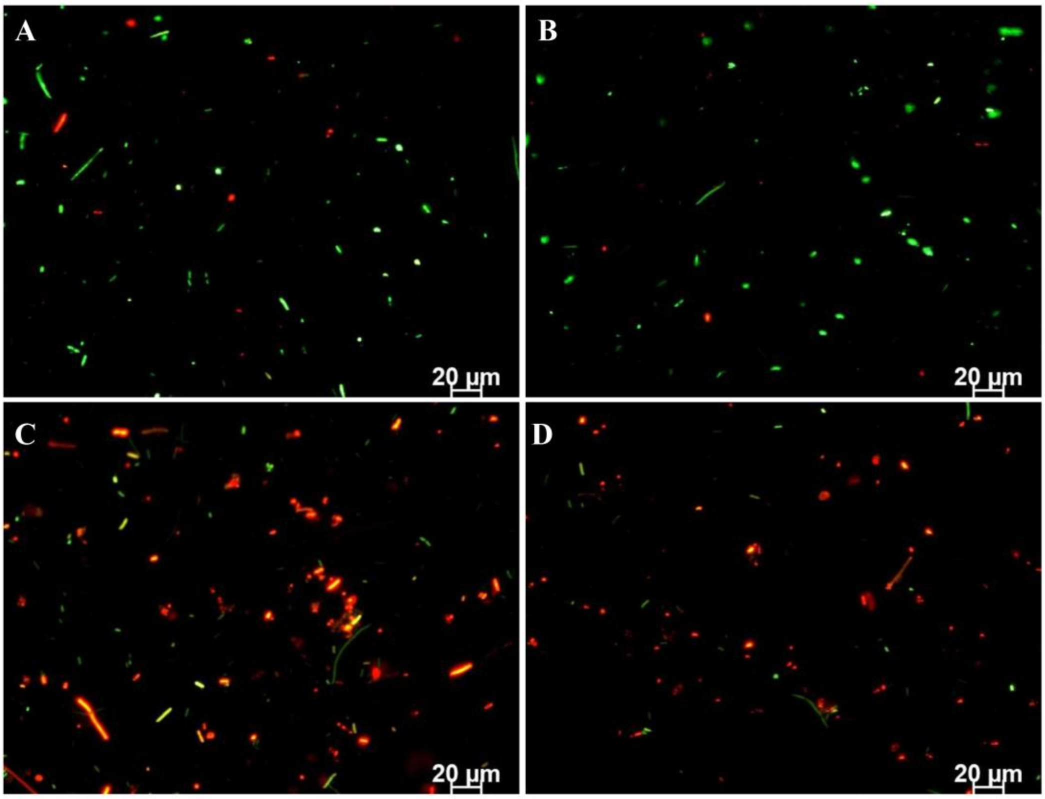

Supplementary Figure S1. (A B) Fluorescent staining of induced DE3: pET-21a. (C D) Fluorescent staining of induced DE3: pET-21a-ORF70.
